# The Abundance of Endofungal Bacterium *Rhizobium radiobacter* (syn. *Agrobacterium tumefaciens*) Increases in Its Fungal Host *Piriformospora indica* during the Tripartite Sebacinalean Symbiosis with Higher Plants

**DOI:** 10.3389/fmicb.2017.00629

**Published:** 2017-04-13

**Authors:** Huijuan Guo, Stefanie P. Glaeser, Ibrahim Alabid, Jafargholi Imani, Hossein Haghighi, Peter Kämpfer, Karl-Heinz Kogel

**Affiliations:** ^1^Institute of Phytopathology, Research Centre for BioSystems, Land Use and Nutrition, Justus-Liebig-University GiessenGiessen, Germany; ^2^Institute of Applied Microbiology, Research Centre for BioSystems, Land Use and Nutrition, Justus-Liebig-University GiessenGiessen, Germany

**Keywords:** *P. Indica*, endofungal bacteria, tripartite symbiosis, endobacteria, plant growth promotion bacteria, endophytes, root colonization

## Abstract

*Rhizobium radiobacter* (syn. *Agrobacterium tumefaciens*, syn. “*Agrobacterium fabrum*”) is an endofungal bacterium of the fungal mutualist *Piriformospora* (syn. *Serendipita*) *indica* (Basidiomycota), which together form a tripartite Sebacinalean symbiosis with a broad range of plants. *R. radiobacter* strain F4 (*Rr*F4), isolated from *P. indica* DSM 11827, induces growth promotion and systemic resistance in cereal crops, including barley and wheat, suggesting that *R. radiobacter* contributes to a successful symbiosis. Here, we studied the impact of endobacteria on the morphology and the beneficial activity of *P. indica* during interactions with plants. Low numbers of endobacteria were detected in the axenically grown *P. indica* (long term lab-cultured, lcPiri) whereas mycelia colonizing the plant root contained increased numbers of bacteria. Higher numbers of endobacteria were also found in axenic cultures of *P. indica* that was freshly re-isolated (riPiri) from plant roots, though numbers dropped during repeated axenic re-cultivation. Prolonged treatments of *P. indica* cultures with various antibiotics could not completely eliminate the bacterium, though the number of detectable endobacteria decreased significantly, resulting in partial-cured *P. indica* (pcPiri). pcPiri showed reduced growth in axenic cultures and poor sporulation. Consistent with this, pcPiri also showed reduced plant growth promotion and reduced systemic resistance against powdery mildew infection as compared with riPiri and lcPiri. These results are consistent with the assumption that the endobacterium *R. radiobacter* improves *P. indica*’s fitness and thus contributes to the success of the tripartite Sebacinalean symbiosis.

## Introduction

Plant-microbe interactions have promoted the development and evolution of land plants. In mutualistic relationships, the plant host benefits from associated microbes by increased mineral nutrient supply, higher tolerance against abiotic stress and increased resistance to pathogens and pests, while the beneficial microbes profit from photosynthates and find protection in the plant’s sphere (Kim et al., 1997; German et al., 2000; Van Wees et al., 2008; Bonfante and Anca, 2009; Pieterse et al., 2009; Breuillin-Sessoms et al., 2015). Rhizobacteria and mycorrhizal fungi are typical examples of mutualistic microbes. For instance, the biological nitrogen fixing *Rhizobium* spp. converts stable nitrogen gas into the biologically useful form ammonia as nitrogen source for the leguminous family (Long, 1989; Parniske, 2000; Oldroyd et al., 2011). Next to their nutritional benefits, arbuscular mycorrhizae fungi (AMF) also induce resistance in plants against necrotrophic pathogens and insects through jasmonic acid and ethylene (JA-ET) dependent signaling pathways (Liu et al., 2007; Pozo and Azcón-Aguilar, 2007). In general, beneficial effects on plants have a high relevance in natural and agricultural ecosystems because of the reduced need of industrial fertilizer in agricultural soils (Yang et al., 2009; Weyens et al., 2009).

Knowledge on the interaction of beneficial fungi and terrestrial plants (bipartite relationships) became even more complex once endofungal bacteria were discovered that form tripartite symbioses with fungi and plants. Endofungal bacteria as symbionts residing in mycelia and spores were first described as Bacteria-Like Organisms (BLOs) in 1970s (Mosse, 1970). Further research showed that such bacteria are either vertically transmitted through vegetative spores, or horizontally transmitted when they are released by the fungal host and subsequently infect newly developed mycelium (Partida-Martinez et al., 2007; Lackner et al., 2009). Endofungal bacteria have been discovered in three clades of beneficial fungi including AMF (Bonfante and Anca, 2009; Naumann et al., 2010; Salvioli et al., 2016), ectomycorrhizal basidiomycetes (Bertaux et al., 2005) and Sebacinalean endophytes (Sharma et al., 2008; Sharma and Kogel, 2009). Bacteria associated with fungi of the genera *Piriformospora* and *Sebacina* belong to two genera of Gram-negative (*Rhizobium* and *Acinetobacter*) and two genera of Gram-positive (*Paenibacillus* and *Rhodococcus*) bacteria (Sharma et al., 2008). Moreover, substantial understanding about the complex role of such bacteria came from the discovery of endobacteria in the rice pathogenic fungus *Rhizopus microsporus* (Partida-Martinez and Hertweck, 2005; Moebius et al., 2014).

*Rhizobium radiobacter* was first discovered in the cytoplasm of the root-colonizing, endophytic Sebacinalean fungus *Piriformospora indica*. The strain *R. radiobacter* F4 (*Rr*F4) was isolated from its fungal host and could be grown in axenic and liquid cultures (Sharma et al., 2008). Using denaturing gradient gel electrophoresis (DGGE) of 16S rRNA gene fragments amplified with universal bacterial 16S rRNA gene targeting primers, one single DNA band was detected in fungal DNA extracts which had the same motility in the DGGE gel as the 16S rRNA gene product amplified from the pure culture of *Rr*F4. These data confirmed that *P. indica* contains a single bacterial species. *Rr*F4 was described as a rod-shaped, Gram-negative bacterium and identified by 16S rRNA gene sequencing and genome comparisons as the Alphaproteobacterium *R. radiobacter* (syn. *Agrobacterium tumefaciens*, syn. “*Agrobacterium fabrum*”; Sharma et al., 2008; Glaeser et al., 2016). Fluorescence *in situ* hybridization (FISH) and PCR amplification detected low numbers of *R. radiobacter* in *P. indica* (Sharma et al., 2008) similar to the ectomycorrhizal *Laccaria bicolor* that contains 1-20 bacteria per fungal cell (Bertaux et al., 2003, 2005). Interestingly, the isolated strain *Rr*F4 and its fungal host *P. indica* showed similar colonization pattern in plant roots as they colonized mainly the maturation zone, and entered into the rhizodermal and cortical layers (Deshmukh et al., 2006; Schäfer et al., 2009; Jacobs et al., 2011; Qiang et al., 2012; Glaeser et al., 2016). In contrast to other endobacteria, the genome size of *Rr*F4 cells is not reduced (Lackner et al., 2011; Fujimura et al., 2014; Naito et al., 2015; Glaeser et al., 2016). The full genome sequencing shows high similarity to the plant pathogenic *A. tumefaciens* (syn. “*Agrobacterium fabrum*”) C58 except vibrant differences in two plasmids, especially the tumor-inducing plasmid (pTi) without T-DNA on it (Goodner et al., 2001; Wood et al., 2001; Lassalle et al., 2011; Slater et al., 2013; Glaeser et al., 2016).

Plant inoculated with *Rr*F4 showed increased shoot and root biomass and pathogen resistance against the powdery mildew fungus *Blumeria graminis* f. sp. *hordei* (*Bgh*) in barley, *Pseudomonas syringae* pv. *tomato* DC3000 (*Pst*) in *Arabidopsis thaliana*, and *Xanthomonas translucens* pv. *translucens* (*Xtt*) in wheat, resembling the beneficial activity induced by *P. indica* (Waller et al., 2005; Sharma et al., 2008; Varma et al., 2012; Oberwinkler et al., 2014; Ye et al., 2014; Glaeser et al., 2016). Moreover, systemic resistance mediated by *Rr*F4, alike *P. indica*, requires a functional jasmonate-based ISR pathway (Stein et al., 2008; Jacobs et al., 2011; Glaeser et al., 2016).

The beneficial effects elicited by *Rr*F4 in inoculated plants suggest that *R. radiobacter* contributes to a successful beneficial symbiosis. Unambiguous elucidation of such a role has been hampered by the fact that all attempts to cure *P. indica* from endobacteria have failed (Glaeser et al., 2016). In the present study, we assessed factors affecting the amount of endofungal bacteria under different growth conditions of fungal cultures. We show that reduced numbers of *P. indica*-associated bacteria lead to changes in the fungal morphology, affects vegetative fungal reproduction, and impedes the biological activity of the fungus. Furthermore, the number of the bacteria especially increases at early stages of root colonization by *P. indica*, suggesting that *R. radiobacter* supports the establishment of the tripartite Sebacinalean symbiosis.

## Materials and Methods

### Plant, Fungal, and Bacterial Materials

Barley (*Hordeum vulgare*) cultivar Golden Promise and *Arabidopsis thaliana* ecotype Columbia-0 (Col-0, N1092) were used. Seeds were surface sterilized and germinated on sterilized filter paper in jars (barley) or germinated on solid ½ Murashige-Skoog (MS) medium supplied with sucrose and solidified with 0.4% gelrite (Arabidopsis).

*Piriformospora indica* DSM 11827 was obtained from the Deutsche Sammlung von Mikroorganismen und Zellkulturen (DSMZ), Braunschweig, Germany. This isolate origins from a sample collected in the Indian Thar desert in 1997 (Verma et al., 1998). The fungus was propagated on modified complete medium (CM, 20 g/L glucose, 2 g/L peptone, 1 g/L yeast extract, 1g/L casamino acids, 1 ml/L 1000× microelements, and 50 ml/L 20 × salt solution) at room temperature (Pham et al., 2004).

*R. radiobacter* F4 is a subculture of strain PABac-DSM isolated from *P. indica* DSM 11827 (Sharma et al., 2008), cultured on YEP medium (5 g/L beef extract, 1 g/L yeast extract, 5 g/L casein hydrolysate, 5 g/L sucrose, and 0.49 g/L MgSO_4_⋅7H_2_O) at room temperature.

### Plant Inoculation

Chlamydospores were collected from 3-week-old *P. indica* cultures, washed and suspended in 0.002% tween-20 with 500,000 chlamydospores per mL. Three-day-old barley seedlings or 7-day-old Arabidopsis seedlings were dip-inoculated in the chlamydospore solution for 1.5 h. Seedlings dipped into tween-20 were used as control. Inoculated barley seedlings were cultured on ½ MS or in pots containing 3:1 mixture of expanded clay (Seramis^®^, Masterfoods) and Oil Dri^®^ (Damolin), fertilized with an aqueous solution of Wuxal Super 8/8/6 (1:1000 v:v; Haug, Düsseldorf, Germany) every week, in a climate chamber under a 16 h photoperiod and 22/18°C day/night (60% rel. humidity, and a photon flux density of 240 μmol m^-2^ s^-1^). Inoculated Arabidopsis seedlings were grown vertically on ½ MS in squared (100 mm) petri dish in climate chamber under short day conditions (8/16 h light/dark, 22/18°C, 60% rel. humidity, and a photon flux density of 183 μmol m^-2^ s^-1^). Roots were harvested at 7 and 14 dpi.

### Re-isolation of *P. indica*

Three-day-old barley seedlings were dip-inoculated with chlamydospores of *P. indica* and cultured on ½ MS medium under sterile condition. Roots were collected after 2 weeks, washed in 70% (v/v) ethanol for 1 min, followed by sodium hypochlorite (3% active chlorine) for 1 min, and cultured on CM agar medium.

### Co-cultivation of *P. indica*

Chlamydospores were collected from 3-week-old axenic *P. indica* cultures and cultured for 3 days in liquid CM medium. For the co-cultivation with GFP-*Rr*F4 (Glaeser et al., 2016), overnight grown GFP-tagged *Rr*F4 was collected, re-suspended and added to the *P. indica* culture with final bacterial concentrations of OD_600_ 0.1, 0.01, and 0.001. For the co-cultivation with plant tissue, *P. indica* cultures were supplemented with root extract and root pieces, respectively, from 7-day-old barley seedlings. Briefly, root extract was collected by grinding 500 mg roots with mortar and pestle in liquid CM medium, followed by passing through a 0.2 μm filter. Root pieces (0.5 cm) were cut from roots with a scissors. The mycelium from each culture was harvested after 7 days to assess the amount of *R. radiobacter* bacteria.

### Production of Protoplasts from *P. indica*

Chlamydospores were collected from 3-week-old *P. indica*, cultured in liquid CM medium with 130 rpm at 28°C for 7 days, harvested by filtering with miracloth and finally washed with 0.9% NaCl solution. The mycelium was crushed with a blender and cultured again for re-generation. After 3 days, the mycelium was filtered, washed, and dissolved in 2% *Trichoderma harzianum* lysing enzyme (Sigma–Aldrich, USA) in SMC buffer (1.33 M sorbitol, 50 mM CaCl_2_, 20 mM MES buffer pH 5.8). After incubating at 37°C for 1 h, 10 mL ice cold STC buffer (1.33 M sorbitol, 50 mM CaCl_2_, 10 mM TrisHCl pH 7.5) was added to stop the enzyme reaction. Protoplasts were collected by centrifugation at 4,000 rpm at 4°C for 10 min, dissolved in 100 μL ice cold STC buffer after washing three times, and then plated on CM agar containing 300 μg/mL spectinomycin and 300 μg/mL ciprofloxacin. After 5 days, single colonies from single protoplasts were picked and transferred to fresh CM agar medium plates containing both antibiotics. After culturing for 3 weeks, DNA was extracted from each fungal sample and used for detection of endobacteria with real-time PCR (qPCR) using specific ITS primers. Chlamydospores were collected from these plates, and used for the second round of propagation. *P. indica* cultures showing no endobacteria in the third round were treated as partially cured *P. indica* cultures (pcPiri).

### Quantification of Endofungal Bacteria by Real-time PCR (qPCR) and DGGE

DNA was extracted with the NucleoSpin^®^ Soil DNA extraction Kit (Macherey-Nagel, Germany) with lysis buffer SL1 according to manufactures’ instructions. DNA concentrations were measured using a NanoDrop ND-1000 (Peqlab Biotechnology, Erlangen, Germany) and adjusted to 25 ng/μL for qPCR analysis. QPCR reactions were performed as described in detail by Glaeser et al. (2016) using a Sybr Green I based detection method. The relative amount of endobacteria was thereby quantified with *Rr*F4 specific 16S rRNA-23S rRNA gene internal transcribed spacer (ITS) targeting primers (ITS_Rhf and ITS_Rhr; Sharma et al., 2008) (**Table 1**). The relative amount of *P. indica* was quantified with *P. indica* translation elongation factor EF-1α Tef targeting primers (Bütehorn et al., 2000) (**Table 1**). Denaturing gradient gel electrophoresis (DGGE) of 16S rRNA gene fragments amplified with universal bacterial 16S rRNA gene targeting primers was performed as described by Glaeser et al. (2010).

**Table 1:** Primers and probes applied in this study.

Primers/Probes	Sequence 5′–3′	Reference
ITS_Rhf	TCAGCACATAACCACACCAATCGCG	Sharma et al., 2008
ITS_Rhr	TGCTTTGTACGCTCGGTAAGAAGGG	Sharma et al., 2008
Tef-f	ACCGTCTTGGGGGTTGTATCC	Bütehorn et al., 2000
Tef-r	TCGTCGCTGTCAACAAGATG	Bütehorn et al., 2000
EUB-338	GCTGCCTCCCGTAGGAGT	Daims et al., 1999
EUB-338-II	GCAGCCACCCGTAGGTGT	Daims et al., 1999
EUB-338-III	GCTGCCACCCGTAGGTGT	Daims et al., 1999
Rh-1247	TCGCTGCCCACTGTG	Ludwig et al., 1998

### Fluorescence *In Situ* Hybridization (FISH) and Staining of Mycelia

Fluorescence *In situ* Hybridization (FISH) was used to detect the endobacterium in fungal materials. Three-week-old *P. indica* and overnight cultured *Rr*F4 were fixed in 50% ethanol for 3–4 h at 4°C, pelleted by centrifugation 5 min with 6,000 rpm at 4°C, and suspended in 1:1 mixture of 1× PBS and 99.9% ethanol after three times washing with PBS. Samples were pipetted on the six recesses-microscope slide (coated with 0.1% gelatin and 0.01% chromium potassium sulfate), dried at 46°C and dehydrated in an increasing ethanol series 50, 80, and 96% (v/v) three min for each. Nine μL hybridization buffer (5 M NaCl, 1 M Tris-HCl, 10% SDS) mixed with 1 μL probe (50 ng/μL) were added to each sample and incubated at 46°C for 1.5 h for hybridization. The hybridization buffer and excess probe were washed with washing buffer (0.5 M EDTA, 1 M Tris-HCl, 10% SDS, 5 M NaCl). Subsequently, the slide was incubated in washing buffer at 48°C for 15 min, and dried at room temperature for microscopic analysis. For staining of fungal DNA, samples were incubated with 10 μL 4’,6-diamidine-2’-phenylindole dihydrochloride (DAPI, Sigma) for 10 min and removed by rinsing with distilled water. The air-dried samples were mounted in AF1 anti-fading reagent (Citifluor Ltd., London, UK), and observed with epi-fluorescence microscope (Leica DM 5000B, Germany). The oligonucleotide probes used in this research were EUB-338-mix, including EUB-338, EUB-338-II, and EUB-338-III for bacteria (Daims et al., 1999), and Rh-1247 for *Rhizobium* (Ludwig et al., 1998) (**Table 1**). The probes were labeled with fluorescein isothiocyanate (FITC, Sigma). The excitation and emission wavelengths were 488 and 530 nm for FITC-labeled EUB-338, and 358 and 461 nm for DAPI staining.

Fluorescent wheat germ agglutinin (WGA) staining was used to detect fungi in barley roots. Root samples were washed in distilled water three times and fixed in fixation solution (chloroform:ethanol:trichloroacetic acid [20%:80%:0.15%]) for 24 h. Subsequently, the materials were treated with 10% KOH for 30 s and washed in 1× PBS buffer (pH 7.4) three times for 5 min. Thereafter, root material was stained in 10 μg/mL WGA solution (Alexa Fluor 488 [WGA_AF488_], Molecular Probes^TM^, ThermoFisher Scientific, Germany) containing 0.02% surfactant Silwet L-77. Vacuum infiltration was applied during the root staining with 25 mm Hg for 1 min. After washing with 1× PBS buffer, root samples were mounted on glass slides for epifluorescence microscopy. WGA_AF488_ was excited with 488-nm and detected at 505–540 nm.

### Biological Activity of *P. indica*

Barley seedlings were dip-inoculated with chlamydospores of *P. indica* and cultured in pots containing 3:1 expanded clay and Oil Dri^®^ (fertilized with Wuxal 8/8/6) for the growth promotion assay. Shoot and root fresh weight (FW) was measured after 3 weeks. Seedlings cultured in pots containing soil were used for the pathogen assay. Detached leaf-segment assays were performed on third leaves of 3-week-old plants with powdery mildew. Leaf segments were plated on water agar (1.5% agar) containing 5% benzimidazole, and inoculated with 15 conidia mm^-2^ of *Blumeria graminis* f. sp. *hordei* (*Bgh* race A6) for 10 min. Pustules were counted with a binocular microscope after 6 days.

## Results

### Detection of Endofungal *R. radiobacter*

**FIGURE 1:**
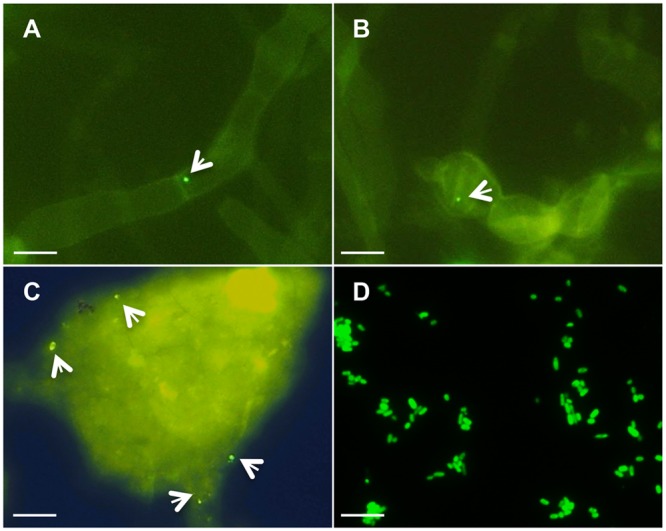
**Detection of endobacteria in *Piriformospora indica* by Fluorescence *In Situ* Hybridization (FISH).** The fungus was grown in liquid complete medium (CM) medium for 3 weeks, and fixed for FISH analysis using the universal bacterial probe EUB-338-FITC (**Table 1**). FISH staining (green signal) detects bacteria in hypha **(A)**, chlamydospores **(B)**, and crushed mycelium **(C)**. **(D)** FISH detection of pure *Rr*F4 cells (positive control). White arrows point to bacteria. Microscopic analyses were done at 1,000-fold magnification; Bars indicated 10 μm.

*Piriformospora indica*-associated endofungal *R. radiobacter* was originally discovered in mycelia by FISH (Sharma et al., 2008). Corroborating this prior result, we detected low numbers of endobacteria in both mycelia and chlamydospores of steadily grown axenic *P. indica* cultures (**Figures 1A,B**). To exclude the possibility that the hyphal wall constituted a barrier for FISH probes and thus conditioned the low number of fluorescent signals, the FISH method was also applied on crushed hyphae (**Figure 1C**) and fungal protoplasts, which confirmed the low abundance of endobacteria. Analysis with a *Rhizobium* specific probe confirmed the results (Supplementary Figure S1). Interestingly, while the endobacterium detected in the mycelium was coccoid-shaped and much smaller in size, isolated *Rr*F4 cells detected by FISH showed rod-shaped cells with a mean size of 1.2–2.0 μm in length and 0.7–0.9 μm in width (**Figure 1D**). Also, consistent with the previous study (Sharma et al., 2008), denaturing gradient gel electrophoresis (DGGE) of 16S rRNA gene fragments amplified with universal bacterial 16S rRNA gene targeting primers detected only one single DNA band in fungal DNA extracts, which had the same motility in the DGGE gel as the 16S rRNA gene product amplified from the pure culture of *Rr*F4 (Supplementary Figure S2).

### The Amount of Endofungal Bacteria Varies with the Type of Fungal Culture

**FIGURE 2:**
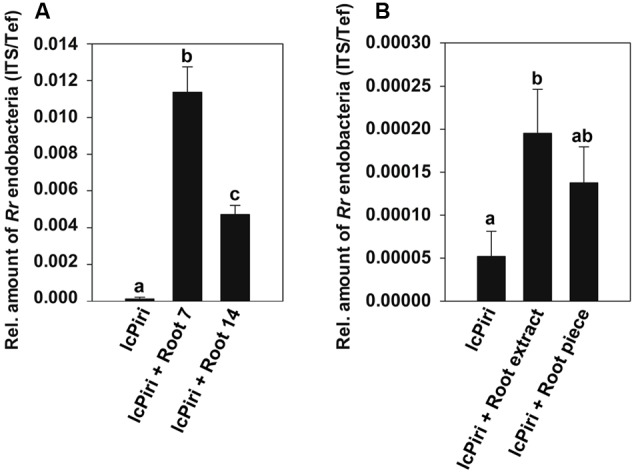
**Quantification of endobacteria in *P. indica*. (A)** Three-day-old barley seedlings were dip-inoculated with chlamydospore solutions and cultured on ½ Murashige-Skoog (MS) in sterile jars. For comparison, chlamydospores (lcPiri) were cultured in liquid CM medium in a flask in the absence of root tissue. Endobacteria were quantified in barley roots (at 7 and 14 dpi) and in 14-day-old liquid cultures. **(B)** Chlamydospores of lcPiri were cultured in liquid CM medium for 3 days. Subsequently, root extracts and root pieces, respectively, from fresh barley roots were added to the cultures. Mycelium from each culture was harvested after 7 days and assessed for endobacteria. Mean values based on three independent biological replicates are given. Different letters on the top of the bars indicate statistically significant differences tested by one-way analysis of variance performed with the Tukey test (*P* < 0.05).

We addressed the question whether the number of endofungal bacteria varies in *P. indica* when grown under different conditions. Therefore, the amount of *R. radiobacter* was quantified in the fungus growing in liquid culture vs. fungus colonizing plant roots. The absolute number of bacteria was calculated with the standard curve for amplification of *Rr*F4’s ITS, while the amount of *P. indica* was calculated using a standard curve based on the amplification of the fungal *Tef* gene (Basiewicz et al., 2012). As indicated by melt curve analysis, only primer dimers could be detected for a standard containing 100 ITS targets per PCR reaction (Supplementary Figure S3). Based on the information that *Rr*F4 has three *rrn* operons (Glaeser et al., 2016) the detection limit of *R. radiobacter* by quantitative RT-PCR is approximately 33 bacteria cells per PCR reaction. Thus, in order to detect endobacterium efficiently, approximately 500 mg mycelium was necessary for DNA extraction. The relative number of bacteria based on the genome ratio between endobacteria and *P. indica* is shown in **Figure 2**. We found increased numbers of *R. radiobacter* in fungal mycelia colonizing barley roots as compared with *P. indica* from liquid cultures (lcPiri). Moreover, the number of bacteria detected in root samples was higher at 7 dpi than at 14 dpi as shown in three independent biological replicates (**Figure 2A**).

Next, we addressed the question whether the number of *R. radiobacter* in mycelia grown in liquid culture was affected by plant derived compounds. For this purpose, a liquid culture started with chlamydospores was supplemented with root segments or extracts of fresh barley roots, respectively, and harvested for *R. radiobacter* quantification after 1 week. qPCR analysis revealed increased relative amounts of bacteria in the supplemented *P. indica* cultures compared with the non-supplemented fungal culture (**Figure 2B**).

### Quantification of Endobacteria in *P. indica* Isolated from Plant Roots

**FIGURE 3:**
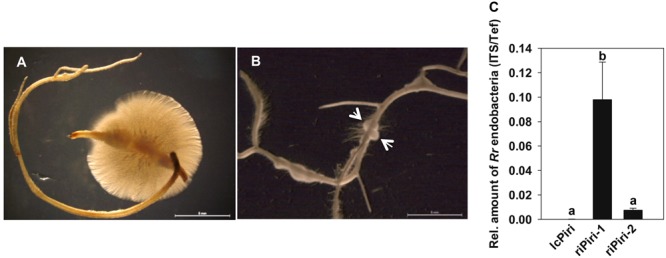
**Re-isolation of *P. indica* from barley roots and quantification of endobacteria.** Seedlings inoculated with lcPiri were cultured on ½ MS in sterile jar for 2 weeks. Thereafter roots were surface-sterilized, cut into small pieces, and cultured on solid or in liquid CM medium, respectively. **(A)** *P. indica* colony developing around a surface-sterilized root piece on CM agar medium. **(B)** *P. indica* colony developing around a surface-sterilized root piece in liquid CM medium. **(C)** Relative amount of endofungal bacteria in cultures of riPiri-1 and sub-culture riPiri-2 was quantified with ITS targets of *Rr*F4 related to the *Tef* gene of *P. indica*. Mean values and standard errors based on three independent biological replicates. Different letters indicate statistically significant differences tested by one-way analysis of variance performed with the Tukey test (*P* < 0.05).

Since the relative amount of bacterial cells increased in *P. indica* during colonization of barley roots, we addressed the question whether the number of bacteria remained high in axenic cultures of *P. indica* when the fungal inoculum was re-isolated from roots. To this end, *P. indica*-colonized barley roots were surface sterilized and subsequently incubated on solid or liquid CM medium. Once fungal hyphae that resided inside the root tissue broke through the root surface, they formed colonies around the root pieces within a week (**Figures 3A,B**). Fungus from this culture was recorded as re-isolated *P. indica* (riPiri-1); it was further transferred and sub-cultured as riPiri-2. As shown in **Figure 3C**, the relative amount of endobacteria increased significantly in riPiri compared to the lcPiri, and the amount in riPiri-1 also was significantly higher than in riPiri-2. These results together show that the amount of *R. radiobacter* increases in the tripartite Sebacinalean symbiosis, whereas it decreases during liquid or axenic culturing of the fungal host in the absence of a plant root. A release of bacterial cells from the *P. indica* cultures was not observed, neither on agar plates nor in liquid medium.

### Antibiotics Reduce the Number of Bacteria in *P. indica*

**FIGURE 4:**
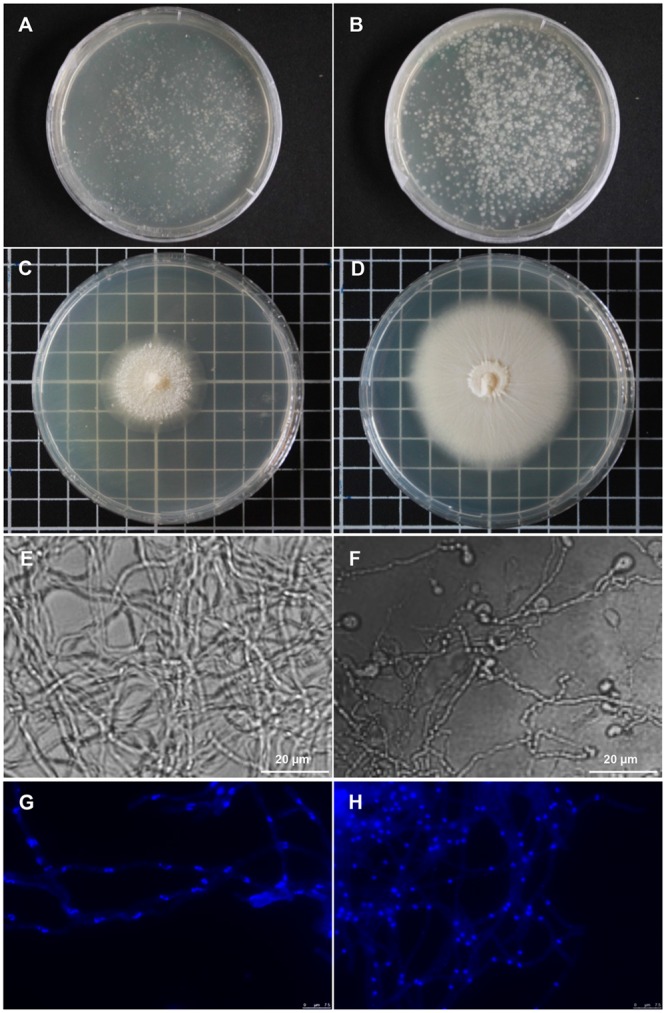
***Piriformospora indica* cultures grown from single protoplasts in the presence of antibiotics. (A)** Fungal colonies regenerated from single protoplasts of lcPiri cultures grown for 5 days on CM medium containing 300 μg/mL spectinomycin and 300 μg/mL ciprofloxacin. **(B)** Colonies grown on CM medium without antibiotics. **(C)** Single colony picked up from A and propagated on medium with the same antibiotics combination for 10 days. **(D)** Single colony picked up from B and propagated without antibiotics for 10 days. **(E)**: Microscopy of *P. indica* mycelium from image C (cultured in the presence of antibiotics). **(F)** Microscopy of the *P. indica* culture from image D (cultured in the absence of antibiotics). Chlamydospores are clearly visible in this culture. **(G,H)** Nuclei were stained with DAPI and show blue fluorescence under the fluorescence microscope. Mycelium from an antibiotics-treated **(G)** and a control culture **(H)**.

While biological activities of both, *P. indica* and the isolated strain *Rr*F4, have been demonstrated many times, it is not known whether *R. radiobacter* is required for a successful Sebacinalean symbiosis (Sharma et al., 2008; Glaeser et al., 2016). To further address this question, we used antibiotics to cure *P. indica* of endofungal bacteria. To this end, fungal protoplasts were treated with antibiotics. After three rounds of protoplastation in the presence of a mixture of 300 μg/ml spectinomycin and 300 μg/ml ciprofloxacin, growth of *P. indica* cultures was delayed compared with the culture from protoplasts that were not treated with antibiotics (**Figures 4A,B**). The diameter of the colonies that derived from single antibiotic-treated protoplasts had a size of 3 cm, while the diameter of control colonies was approximately 6 cm (**Figures 4C,D**). No apparent alterations in morphology of fungal hyphae were observed, while formation of vegetative chlamydospores was clearly reduced in *P. indica* cultures under antibiotics selection (**Figures 4E,F**). Moreover, DAPI staining revealed differences in the shape of fungal nuclei: hyphae of antibiotics-treated *P. indica* culture had rod-shaped nuclei (**Figure 4G**), while spherical-shaped nuclei were observed in non-treated mycelia (**Figure 4H**). Significantly, no endobacteria could be detected by qPCR in antibiotics-treated *P. indica* cultures. However, prolonged fungal cultivation in the absence of antibiotics resulted in the recovery of a low number of endobacterial cells.

### Re-introduction of *Rr*F4 Cells into *P. indica* Failed

**FIGURE 5:**
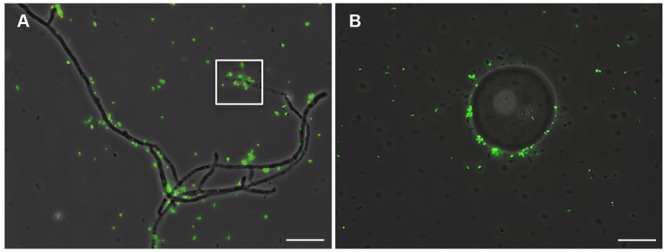
**GFP-tagged *Rr*F4 sticking around *P. indica*.** Chlamydospores of *P. indica* were germinated in liquid CM medium for 3 days, before a GFP-*Rr*F4 suspension was added to the culture. **(A)** GFP-tagged *Rr*F4 stacking around the hyphae. The square box showed GFP-tagged *Rr*F4 around a hyphal tip. **(B)** GFP-tagged *Rr*F4 at the surface of chlamydospores. Bars indicated 10 μm.

GFP-tagged *Rr*F4 cells were co-cultured with *P. indica* to address the question whether isolated *Rr*F4 can invade fungal hyphae. GFP-tagged *Rr*F4 cells were added to a three-day-old liquid culture of *P. indica* started from chlamydospores. As shown in the **Figure 5**, many GFP-tagged *Rr*F4 cells stuck around the hyphae (**Figure 5A**) and the surface of chlamydospores (**Figure 5B**), while they were not detected inside these structures. As the fungal wall may constitute a barrier for bacteria, protoplasts of *P. indica* were also incubated with GFP-tagged *Rr*F4, but this strategy could neither improve the uptake of bacteria (data not shown).

### Biological Activity Exhibited by riPiri, pcPiri, and lcPiri

**FIGURE 6:**
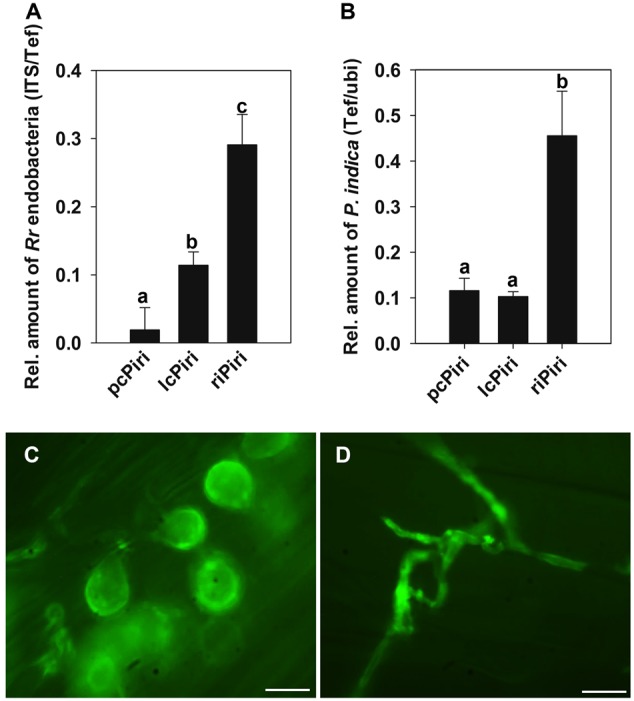
**Colonization of barley roots by riPiri, lcPiri, and pcPiri.** Three-day-old seedlings were dip-inoculated with respective mycelia. Seedlings were grown in soil in a growth chamber and harvested after 1 week for quantification and WGA-staining. **(A)** Relative amount of *R. radiobacter* bacteria based on the genome ratio of *RrF4* vs. *P. indica* on barley roots. **(B)** Quantification of the amount of *P. indica* mycelium on barley roots. **(C)** Pear-shaped chlamydospores and mycelium in a root inoculated with riPiri. **(D)** Mycelium in a root inoculated with pcPiri. Mean values and standard errors of three independent biological replicates are given. Different letters on the top of the bars indicate statistically significant differences tested by one-way analysis of variance performed with the Tukey test (*p* < 0.05). Bars indicated 10 μm.

To answer the question whether *P. indica* cultures that contain different amount of endobacteria exhibit comparable biological activities, biomass and systemic resistance of barley seedlings were measured upon inoculation with riPiri, pcPiri, and lcPiri, respectively. By 7 dpi, roots were checked for fungal colonization and the amount of endobacteria. The *Tef* gene of *P. indica* was used for the quantification of the fungus, while the specific ITS primer system was used for the quantification of *R. radiobacter* bacteria. We found that the relative amount of endobacteria in root samples colonized by riPiri was significantly higher compared with roots colonized by lcPiri and pcPiri (**Figure 6A**). Yet, that bacteria were detectable in root samples colonized by pcPiri shows that single protoplast cultivation and antibiotics treatment did not completely cure *P. indica* from *R. radiobacter*. riPiri also showed the highest colonization density on barley roots as compared with pcPiri and lcPiri (**Figure 6B**). Consistent with this finding, high amounts of pear-shaped chlamydospores and mycelium were observed in root inoculated with riPiri when roots were stained with chitin-specific WGA-Alexa Fluor 488 to visualize mycelia (**Figure 6C**), while less colonization was seen in roots inoculated with pcPiri (**Figure 6D**). Importantly, less chlamydospores were seen in mycelia of lcPiri and most significantly in pcPiri, suggesting that vegetative reproduction is negative affected when the number of bacteria is limited.

**FIGURE 7:**
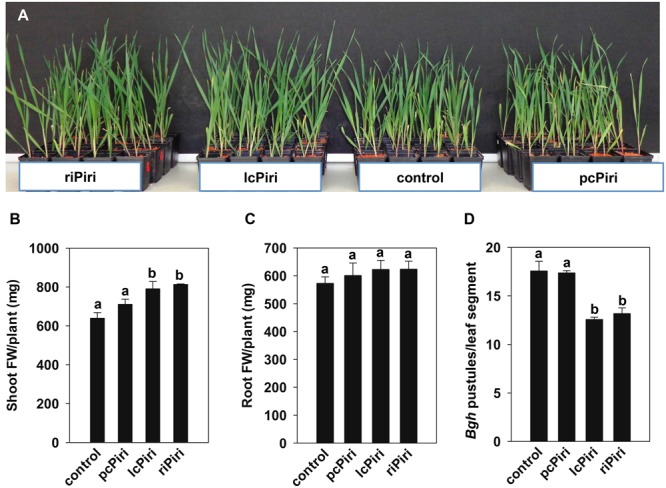
**Biological activity conferred by pcPiri, lcPiri and riPiri on barley.** Three-week-old plants inoculated with riPiri, lcPiri, pcPiri, and non-inoculated control plants were used for biomass assessments and a pathogen assay. **(A)** Shoot fresh weights (FW); **(B)** Root FW. **(C)** Number of *Bgh* pustules on detached leaves. **(D)** Three-week-old barley plants treated with *P. indica* cultures. Bars indicate standard errors based on three independent biological replicates. Letters on the top of the bars indicate statistically significant differences tested by one-way analysis of variance performed with the Tukey test (*p* < 0.05).

Next, we assessed fungal growth promotion activity. Three-week-old barley plants inoculated with riPiri, lcPiri, and pcPiri were harvested for biomass analysis. Compared with non-inoculated plants, plants treated with either fungal culture showed some growth promotion (**Figure 7A**). Accordingly, the shoot FW of colonized plants was always higher compared to non-colonized control plants, though it was increased significantly only in seedlings treated with riPiri (17.7%) and lcPiri (15.9%) (**Figure 7B**). Moreover, root FW slightly increased in all colonized plants though the effect was not significant (**Figure 7C**). We also assessed the resistance-inducing activity of *P. indica* cultures against *Bgh* in barley. Barley seedlings were dip-inoculated with crushed *P. indica* cultures. Three weeks later, third leaves were harvested and inoculated with *Bgh* conidia in a detached leaf assay to assess powdery mildew resistance. The pustules on leaves from plants inoculated with riPiri and lcPiri were equally reduced by 21% compared to control plants, while there was no significant difference for plants infected with pcPiri (2.3%) compared with untreated controls (**Figure 7D**).

**FIGURE 8:**
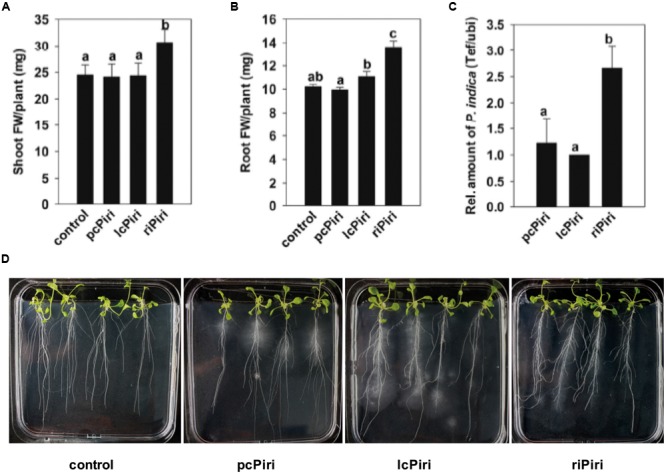
**Biological activity conferred by pcPiri, lcPiri, and riPiri on Arabidopsis.** Seedlings were dip-inoculated with *P. indica* cultures and incubated on petri dishes. **(A)** Shoot FW; **(B)** Root FW; **(C)** Relative amount of *P. indica* in Arabidopsis roots (colonization density). **(D)** Root structure (primary and secondary root formation) of 3-week-old Arabidopsis seedlings. Bars indicate standard errors based on three independent biological replicates. Letters on the top of the bars indicate statistically significant differences tested by one-way analysis of variance performed with the Tukey test (*p* < 0.05).

We also assessed the biological activity of the *P. indica* cultures in Arabidopsis (**Figure 8**). Consistent with the above data, treatment of Arabidopsis seedlings with riPiri resulted in a statistically significant increase in shoot and root FWs. Moreover, riPiri colonized Arabidopsis roots much stronger than lcPiri and pcPiri, which is consistent with the hypothesis that bacteria support the fitness of the endophytic fungus.

## Discussion

### *R. radiobacter* in *Piriformospora indica*

In the present work, we show a positive correlation between the number of endofungal *R. radiobacter* that are associated with the fungal mutualist *P. indica* and the biological activity the mutualist exerts on plants. Furthermore, there is an indication for *P. indica*’s requirement for endobacteria for full vegetative propagation via chlamydospores. Endobacteria were detected by independent methods including FISH, qPCR, and sub-culturing of the isolated strain *Rr*F4. In continuous fungal cultures the endobacteria reach very low numbers. This low amount and limited detection have been the bottlenecks for the analysis of the role endobacterium in the tripartite Sebacinalean symbiosis. The finding that the amount of endofungal bacteria increased when *P. indica* colonized roots opened new possibility to study endofungal bacteria’s role in the tripartite symbiosis. The higher amount of bacteria in riPiri that was isolated directly from barley roots, and the steady decrease during axenic subculture suggest that a plant host promotes the propagation of the endofungal bacterium in its host fungus. Similar observation is known from the fungus *Gigaspora margarita*, in which the expression of the *ftsZ* gene of the endocellular bacterium *Candidatus* Glomeribacter gigasporarum was up-regulated when the fungal host colonized on plant (Anca et al., 2009).

### Endobacteria Influence the Fitness of *P. indica*

In order to generate *P. indica* mycelia with substantially lowered numbers of bacterial cells, we combined strategies recently published to remove endobacteria from its fungal hosts. A bacteria-free fungus *Rhizopus microspores* was obtained through antibiotics treatment (Partida-Martinez and Hertweck, 2005), while repeated passages through single-spore inoculation of plants was used to dilute the initial *Candidatus* Glomeribacter gigasporarum (*CaGg*) population in the spores of the AM fungus *Gigaspora margarita* (Lumini et al., 2007). We show here that a combination of single fungal protoplast propagation and antibiotics treatment at least transiently reduced the number of active bacterial cells in *P. indica*. *P. indica* depleted in *R. radiobacter* showed delayed germination from chlamydospores, reduced growth in axenic cultures and less sporulation. Although we cannot exclude that the effect comes from antibiotics treatment, those morphological changes could be induced by the reduced number of endobacteria. Because there was no morphology change at the beginning of antibiotics treatment, those changes only happen after several rounds of selection. That endofungal bacteria support the fitness of its fungal host has been demonstrated with pathogenic and mutualistic fungi. Cured *Rhizopus microspores* show no sporulation (Partida-Martinez and Hertweck, 2005; Partida-Martinez et al., 2007), cured fungus *Gigaspora margarita* shows limited changes in spore morphology, while the symbiosis with this endobacterium *CaGg* increased the environmental fitness and bioenergetics potential of the AM fungal host (Lumini et al., 2007; Salvioli et al., 2016). *P. indica* cultures treated with antibiotics (pcPiri) initially seemed to be completely cured from bacteria. However, when inoculated to barley seedlings, bacteria were again detected in plant root samples. However, the relative amount of bacteria was significantly reduced compared with root samples colonized by steadily grown *P. indica*. We concluded that antibiotics-treated *P. indica* culture was only partially cured from endobacteria, and the fitness and asexual reproduction of the fungal host is compromised with reduced endobacteria.

### Endobacteria Exhibit Beneficial Activity on Plant Hosts

The colonization and induced biological activity were compared among three types of *P. indica* cultures. The determination of the genome ratio of *R. radiobacter* vs. *P. indica* in plant roots inoculated with pcPiri, lcPiri, and riPiri confirmed the differences among these three *P. indica* cultures with respect to the presence of endobacteria. The riPiri culture contained the highest number of endobacteria, while pcPiri contained the lowest number. The riPiri culture showed most efficient colonization of barley and Arabidopsis roots with high amounts of mycelium and chlamydospores. These results support the hypothesis that the amount of endobacteria had an effect on both fungal colonization and sporulation. Compared with control plants, plants either treated with riPiri or lcPiri, respectively, had significantly increased shoot weights, which is consistent with earlier findings that *P. indica* promotes plant growth (Waller et al., 2005; Sharma et al., 2008; Qiang et al., 2012). Although the increase in bacterial cell numbers in riPiri was significant, numbers were still low as shown by qPCR and confirmed by low numbers of cells detected by FISH analysis (data not shown). Even a small but significant increase in *R. radiobacter* cell numbers has an effect on the biological activity of *P. indica*. The mechanism behind the activation of the fungal activity is not resolved. Genome analysis of the endobacteria *Candidatus* Glomeribacter gigasporarum in *Gigaspora margarita* indicated that the endobacterium could provide vitamin B12 to the fungal host and plant host (Ghignone et al., 2012). The same genes for the synthesis pathway of vitamin B12 were also present in the genome of *Rr*F4 (Glaeser et al., 2016). This indicates that *Rr*F4 may also support *P. indica* with vitamine B12. Salvioli et al. (2016) showed that the endofungal bacteria can increased the fitness of root colonizing fungi and thereby may affects their colonization efficiency and biological activity. Taken together, these data are consistent with the hypothesis that the endobacterium mediates growth promotion of plants and thus contributes to the biological activity induced by its fungal host *P. indica*. Whether the effects of the endobacteria on the plant growth are direct or indirect (by activating *P. indica*) still needs further investigations.

### Cultured *Rr*F4 Cells Do Not Invade the Fungus

The co-culture of GFP-tagged *Rr*F4 and *P. indica* showed GFP-*Rr*F4 cells sticking around the hyphae and chlamydospores instead of entry into hyphae. Type 2 secretion system (T2SS) in endosymbiont *Burkholderia* was previously shown to be central for the active invasion into living fungus *Rhizopus*, because T2SS releases chitinase, chitosanase and chitin-binding proteins to soften the cell wall of fungus and allow the bacterial entry into fungal hyphae (Moebius et al., 2014). Based on this information, we checked the genome sequence of *Rr*F4. Not surprisingly, neither the whole gene cluster that is responsible for the encoding of T2SS components nor the genes synthesizing chitinase and chitosanase is existing in the genome of *Rr*F4 (Glaeser et al., 2016). Instead *Rr*F4 has a type IV secretion system, which is a remarkable characteristic in *Agrobacterium* and mediates the transfer of plasmid DNA fragment into plant genome (Sharma et al., 2008; Fronzes et al., 2009; Glaeser et al., 2016). The missing responsible genes and T2SS in *Rr*F4 could be the reason for the unsuccessful invasion of *Rr*F4 into *P. indica*. However, protoplastation of hyphae and incubation with the *Rr*F4 cells also did not result in bacterial uptake indicating that additional factors may be required for bacterial transfer. Our co-culture experiment showed that *Rr*F4 sticking around the fungus including the tip of hyphae and spores, which are the active part from *P. indica.* Those hyphae tip and germinating spores can be potential entry sites for bacteria.

### Endobacteria *Rhizobium* in a VBNC State

Some Gram-negative bacteria have extraordinary ability to survive under harsh environment. They enter into a specific growth stage of low metabolic activity, the viable but non-culturable (VBNC) state, which is similar to a stationary growth phase in a lab culture. Bacteria, including *Agrobacterium* and *Rhizobium* species (Alexander et al., 1999), enter the VBNC state if the environment is not sufficient enough to keep steady growth (Llorens et al., 2010). In the VBNC state, bacterial cells perform morphological and physiological adaptations to the changed conditions. The bacterial cells become smaller as the result of reductive division and dwarfing, and more resistant against different kinds of harm because of the formation of cell envelopes (Nyström, 2004; Llorens et al., 2010). Given that bacteria decreased in abundance and changed to the small spherical shape (compared to the rod-shaped cells in pure culture) as shown by FISH and Sybr Green I straining, we assume that *R. radiobacter* switched into the VBNC state in axenic long-term *P. indica* lab-cultures. In this VBNC state, bacterial cells are more resistant to antibiotics, which could be one reason for the insufficient antibiotics treatment to cure endobacteria from their fungal host. Because the VBNC cells are less metabolically active, the concentration of ribosomes decreased. This may have resulted in a reduced detection efficiency by FISH. Our data are consistent with the hypothesis that bacteria resuscitate from the VBNC to become metabolically active and growing cells in the presence of the plant or plant-derived extracts which may trigger the activity of the bacterium by, e.g., supplying it with enough nutrition and better propagation conditions. This could explain the increased amount of *R. radiobacter* when *P. indica* colonized plant roots.

## Conclusion

The data presented here support the hypothesis that the endobacterium of *P. indica* regulates the fitness and sporulation of *P. indica*, influences the colonization on plant roots, and further contributes to the biological activity induced by *P. indica*. There is robust association between endobacteria and *P. indica*, since *P. indica* cannot be fully cured from the endobacterium, while free *Rr*F4 did not re-enter mycelium. Our studies suggest *R. radiobacter* contributes to the tripartite Sebacinalean symbiosis but further studies are needed to fully elucidate the mechanism exerted by the endofungal bacteria during the mutualistic tripartite Sebacinalean interactions.

## Author Contributions

HG, SG, JI, and K-HK designed the research. HG, IA, and HH performed the experiments. HG, SG, JI, and K-HK analyzed data. K-HK and PK got the funding. HG, SG, and K-HK wrote the manuscript.

## Conflict of Interest Statement

The authors declare that the research was conducted in the absence of any commercial or financial relationships that could be construed as a potential conflict of interest.

## References

[B1] Alexander E. Pham D. Steck T. R. (1999). The viable-but-nonculturable condition is induced by copper in *Agrobacterium tumefaciens* and *Rhizobium leguminosarum*. *Appl. Environ. Microbiol.* 65 3754–3756. 10427081 10.1128/aem.65.8.3754-3756.1999 PMC91566

[B2] Anca I. A. Lumini E. Ghignone S. Salvioli A. Bianciotto V. Bonfante P. (2009). The *ftsZ* gene of the endocellular bacterium ‘*Candidatus* Glomeribacter gigasporaum’ is preferentially expressed during the symbiotic phases of its host mycorrhizal fungus. *Mol. Plant Microbe Interact.* 22 302–310. 10.1094/MPMI-22-3-0302 19245324

[B3] Basiewicz M. Weiß M. Kogel K. H. Langen G. Zorn H. Zuccaro A. (2012). Molecular and phenotypic characterization of *Sebacina vermifera* strains associated with orchids, and the description of *Piriformospora williamsii* sp. nov. *Fungal Biol.* 116 204–213. 10.1016/j.funbio.2011.11.003 22289766

[B4] Bertaux J. Schmid M. Hutzler P. Hartmann A. Garbaye J. Frey-Klett P. (2005). Occurrence and distribution of endobacteria in the plant-associated mycelium of the ectomycorrhizal fungus *Laccaria bicolor* S238N. *Environ. Microbiol.* 7 1786–1795. 10.1111/j.1462-2920.2005.00867.x 16232293

[B5] Bertaux J. Schmid M. Prevost-Bourre N. C. Churin J. L. Hartmann A. Garbaye J. (2003). In situ identification of intracellular bacteria related to *Paenibacillus* spp. in the mycelium of the ectomycorrhizal fungus *Laccaria bicolor* S238N. *Appl. Environ. Microbiol.* 69 4243–4248. 10.1128/AEM.69.7.4243-4248.2003 12839806 PMC165139

[B6] Bonfante P. Anca I. A. (2009). Plant, mycorrhizal fungi, and bacteria: a network of interactions. *Annu. Rev. Microbiol.* 63 363–383. 10.1146/annurev.micro.091208.073504 19514845

[B7] Breuillin-Sessoms F. Floss D. S. Gomez K. Pumplin N. Ding Y. Levesque-Tremblay V. (2015). Suppression of arbuscule degeneration in *Medicago truncatula phosphate transporter4* mutants is dependent on the ammonium transporter 2 family protein AMT2;3. *Plant Cell* 27 1352–1366. 10.1105/tpc.114.131144 25841038 PMC4558683

[B8] Bütehorn B. Rhody D. Franken P. (2000). Isolation and characterisation of *Pitef1* encoding the translation elongation factor EF-1a of the root endophyte *Piriformospora indica*. *Plant Biol.* 2 687–692. 10.1055/s-2000-16647

[B9] Daims H. Brühl A. Amann R. Schleifer K. H. Wagner M. (1999). The domain-specific probe EUB338 is insufficient for the detection of all *Bacteria*: development and evaluation of a more comprehensive probe set. *Syst. Appl. Microbiol.* 22 434–444. 10.1016/S0723-2020(99)80053-8 10553296

[B10] Deshmukh S. Hückelhoven R. Schäfer P. Imani J. Sharma M. Weiss M. (2006). The root endophytic fungus *Piriformospora indica* requires host cell death for proliferation during mutualistic symbiosis with barley. *Proc. Natl. Acad. Sci. U.S.A.* 103 18450–18457. 10.1073/pnas.0605697103 17116870 PMC1697795

[B11] Fronzes R. Christie P. J. Waksman G. (2009). The structural biology of type IV secretion systems. *Nat. Rev. Microbiol.* 7 703–714. 10.1038/nrmicro2218 19756009 PMC3869563

[B12] Fujimura R. Nishimura A. Ohshima S. Sato Y. Nishizawa T. Oshima K. (2014). Draft genome sequence of the betaproteobacterial endosymbiont associated with the fungus *Mortierella elongata* FMR23-6. *Genome Announc.* 2 e01272–14. 10.1128/genomeA.01272-14 PMC4263831 25502669

[B13] German M. A. Burdman S. Okon Y. Kigel J. (2000). Effects of *Azospirillum brasilense* on root morphology of common bean (*Phaseolus vulgaris* L.) under different water regimes. *Biol. Fertil. Soils* 32 259–264. 10.1007/s003740000245

[B14] Ghignone S. Salvioli A. Anca I. Lumini E. Ortu G. Petiti L. (2012). The genome of the obligate endobacterium of an AM fungus reveals an interphylum network of nutritional interactions. *ISME J.* 6 136–145. 10.1038/ismej.2011.110 21866182 PMC3246228

[B15] Glaeser S. P. Grossart H. P. Glaeser J. (2010). Singlet oxygen, a neglected but important environmental factor: short-term and long-term effects on bacterioplankton composition in a humic lake. *Environ. Microbiol.* 12 3124–3136. 10.1111/j.1462-2920.2010.02285.x 20636377

[B16] Glaeser S. P. Imani J. Alabid I. Guo H. Kumar N. Kämpfer P. (2016). Non-pathogenic *Rhizobium radiobacter* F4 deploys plant beneficial activity independent of its host *Piriformospora indica*. *ISME J.* 10 871–884. 10.1038/ismej.2015.163 26495996 PMC4796927

[B17] Goodner B. Hinkle G. Gattung S. Miller N. Blanchard M. Qurollo B. (2001). Genome sequence of the plant pathogen and biotechnology agent *Agrobacterium tumefaciens* C58. *Science* 294 2323–2328. 10.1126/science.1066803 11743194

[B18] Jacobs S. Zechmann B. Molitor A. Trujillo M. Petutschnig E. Lipka V. (2011). Broad-spectrum suppression of innate immunity is required for colonization of Arabidopsis roots by the fungus *Piriformospora indica*. *Plant Physiol.* 156 726–740. 10.1104/pp.111.176446 21474434 PMC3177271

[B19] Kim K. Y. Jordan D. McDonald G. A. (1997). Effect of phosphate-solubilizing bacteria and vesicular-arbuscular mycorrhizae on tomato growth and soil microbial activity. *Biol. Fertil. Soils* 26 79–87. 10.1007/s003740050347

[B20] Lackner G. Moebius N. Partida-Martinez L. Hertweck C. (2011). Complete genome sequence of *Burkholderia rhizoxinica*, an endosymbiont of *Rhizopus microsporus*. *J. Bacteriol.* 193 783–784. 10.1128/JB.01318-10 21131495 PMC3021220

[B21] Lackner G. Partida-Martinez L. P. Hertweck C. (2009). Endofungal bacteria as producers of mycotoxins. *Trends Micorbiol.* 17 570–576. 10.1016/j.tim.2009.09.003 19800796

[B22] Lassalle F. Campillo T. Vial L. Baude J. Costechareyre D. Chapulliot D. (2011). Genomic species are ecological species as revealed by comparative genomics in *Agrobacterium tumefaciens*. *Genome Biol. Evol.* 3 762–781. 10.1093/gbe/evr070 21795751 PMC3163468

[B23] Liu J. Maldonado-Mendoza I. Lopez-Meyer M. Cheung F. Town C. D. Harrison M. J. (2007). Arbuscular mycorrhizal symbiosis is accompanied by local and systemic alterations in gene expression and an increase in disease resistance in the shoots. *Plant J.* 50 529–544. 10.1111/j.1365-313X.2007.03069.x 17419842

[B24] Llorens J. M. N. Tormo A. Martínez-García E. (2010). Stationary phase in gram-negative bacteria. *FEMS Microbiol. Rev.* 34 476–495. 10.1111/j.1574-6976.2010.00213.x 20236330

[B25] Long S. R. (1989). Rhizobium-legume nodulation: life together in the underground. *Cell* 56 203–214. 10.1016/0092-8674(89)90893-3 2643474

[B26] Ludwig W. Amann R. Martinez-Romero E. Schönhuber W. Bauer S. Neef A. (1998). rRNA based identification systems for rhizobia and other bacteria. *Plant Soil* 204 1–9.

[B27] Lumini E. Bianciotto V. Jargeat P. Novero M. Salvioli A. Faccio A. (2007). Presymbiotic growth and sporal morphology are affected in the arbuscular mycorrhizal fungus *Gigaspora margarita* cured of its endobacteria. *Cell. Microbiol.* 9 1716–1729. 10.1111/j.1462-5822.2007.00907.x 17331157

[B28] Moebius N. Üzüm Z. Dijksterhuis J. Lackner G. Hertweck C. (2014). Active invasion of bacteria into living fungal cells. *eLife* 3:e03007 10.7554/eLife.03007 PMC4166002 25182414

[B29] Mosse B. (1970). Honey-coloured, sessile Endogone spores: II. Changes in fine structure during spore development. *Arch. Microbiol.* 74 129–145. 10.1007/BF00446901

[B30] Naito M. Morton J. B. Pawlowska T. E. (2015). Minimal genomes of mycoplasma-related endobacteria are plastic and contain host-derived genes for sustained life within Glomeromycota. *Proc. Natl. Acad. Sci. U.S.A.* 112 7791–7796. 10.1073/pnas.1501676112 25964324 PMC4485128

[B31] Naumann M. Schüßler A. Bonfante P. (2010). The obligate endobacteria of arbuscular mycorrhizal fungi are ancient heritable components related to the *Mollicutes*. *ISME J.* 4 862–871. 10.1038/ismej.2010.21 20237515

[B32] Nyström T. (2004). Stationary-phase physiology. *Annu. Rev. Microbiol.* 58 161–181. 10.1146/annurev.micro.58.030603.123818 15487934

[B33] Oberwinkler F. Riess K. Bauer R. Garnica S. (2014). Morphology and molecules: the Sebacinales, a case study. *Mycol. Prog.* 13 445–470. 10.1007/s11557-014-0983-1

[B34] Oldroyd G. E. D. Murray J. D. Poole P. S. Downie J. A. (2011). The rules of engagement in the legume-rhizobial symbiosis. *Annu. Rev. Genet.* 45 119–144. 10.1146/annurev-genet-110410-132549 21838550

[B35] Parniske M. (2000). Intracellular accommodation of microbes by plants: a common developmental program for symbiosis and disease? *Curr. Opin. Plant Biol.* 3 320–328. 10.1016/S1369-5266(00)00088-1 10873847

[B36] Partida-Martinez L. P. Hertweck C. (2005). Pathogenic fungus harbours endosymbiotic bacteria for toxin production. *Nature* 437 884–888. 10.1038/nature03997 16208371

[B37] Partida-Martinez L. P. Monajembashi S. Greulich K. O. Hertweck C. (2007). Endosymbiont-dependent host reproduction maintains bacterial-fungal mutualism. *Curr. Biol.* 17 773–777. 10.1016/j.cub.2007.03.039 17412585

[B38] Pham G. H. Kumari R. Singh A. Malla R. Prasad R. Sachdev M. (2004). *Axenic Culture of Symbiotic Fungus Piriformospora indica. Plant Surface Microbiology.* Berlin: Springer-Verlag 593–613.

[B39] Pieterse C. M. J. Leon-Reyes A. Van der Ent S. Van Wees S. C. M. (2009). Networking by small-molecule hormones in plant immunity. *Nat. Chem. Biol.* 5 308–316. 10.1038/nchembio.164 19377457

[B40] Pozo M. J. Azcón-Aguilar C. (2007). Unraveling mycorrhiza-induced resistance. *Curr. Opin. Plant Biol.* 10 393–398. 10.1016/j.pbi.2007.05.004 17658291

[B41] Qiang X. Weiss M. Kogel K. H. Schäfer P. (2012). *Piriformospora indica*-a mutualistic basidiomycete with an exceptionally large plant host range. *Mol. Plant Pathol.* 13 508–518. 10.1111/j.1364-3703.2011.00764.x 22111580 PMC6638644

[B42] Salvioli A. Ghignone S. Novero M. Navazio L. Venice F. Bagnaresi P. (2016). Symbiosis with an endobacterium increases the fitness of a mycorrhizal fungus, raising its bioenergetics potential. *ISME J.* 10 130–144. 10.1038/ismej.2015.91 26046255 PMC4681866

[B43] Schäfer P. Pfiffi S. Voll L. M. Zajic D. Chandler P. M. Waller F. (2009). Manipulation of plant innate immunity and gibberellin as factor of compatibility in the mutualistic association of barley roots with *Piriformospora indica*. *Plant J.* 59 461–474. 10.1111/j.1365-313X.2009.03887.x 19392709

[B44] Sharma M. Kogel K. H. (2009). “Fungal isolates of the order *Sebacinales* provide growth promotion and systemic disease resistance to barley,” in *Biological Control of Fungal and Bacterial Plant Pathogens* Vol. 43 eds Elad Y. Maurhofer M. Keel C. Gessler C. Duffy B. (Dijon: IOBC/wprs Bull) 211–215.

[B45] Sharma M. Schmid M. Rothballer M. Hause G. Zuccaro A. Imani J. (2008). Detection and identification of bacteria intimately associated with fungi of the order Sebacinales. *Cell Microbiol.* 10 2235–2246. 10.1111/j.1462-5822.2008.01202.x 18637023

[B46] Slater S. Setubal J. C. Goodner B. Houmiel K. Sun J. Kaul R. (2013). Reconciliation of sequence data and updated annotation of the genome of *Agrobacterium tumefaciens* C58, and distribution of a linear chromosome in the genus *Agrobacterium*. *Appl. Environ. Microbiol.* 79 1414–1417. 10.1128/AEM.03192-12 23241979 PMC3568625

[B47] Stein E. Molitor A. Kogel K. H. Waller F. (2008). Systemic resistance in Arabidopsis conferred by the mycorrhizal fungus *Piriformospora indica* requires jasmonic acid signalling and the cytoplasmic function of NPR1. *Plant Cell Physiol.* 49 1747–1751. 10.1093/pcp/pcn147 18842596

[B48] Van Wees S. C. M. Van der Ent S. Pieterse C. M. J. (2008). Plant immune responses triggered by beneficial microbes. *Curr. Opin. Plant Biol.* 11 443–448. 10.1016/j.pbi.2008.05.005 18585955

[B49] Varma A. Bakshi M. Lou B. Hartmann A. Oelmueller R. (2012). *Piriformospora indica*: a novel plant growth promoting mycorrhizal fungus. *Agric. Res.* 1 117–131. 10.1007/s40003-012-0019-5

[B50] Verma S. Varma A. Rexe K. H. Hassel A. Kost G. Sarbhoy A. (1998). *Piriformospora indica*, gen. et sp. nov., a new root-colonizing fungus. *Mycologia* 90 896–903. 10.2307/3761331

[B51] Waller F. Achatz B. Baltruschat H. Fodor J. Becker K. Fischer M. (2005). The endophytic fungus *Piriformospora indica* reprograms barley to salt-stress tolerance, disease resistance, and higher yield. *Proc. Natl. Acad. Sci. U.S.A.* 102 13386–13391. 10.1073/pnas.0504423102 16174735 PMC1224632

[B52] Weyens N. van der Lelie D. Taghavi S. Newman L. Vangronsveld J. (2009). Exploiting plant-microbe partnerships to improve biomass production and remediation. *Trends Biotechnol.* 27 591–598. 10.1016/j.tibtech.2009.07.006 19683353

[B53] Wood D. W. Setubal J. C. Kaul R. Monks D. E. Kitajima J. P. Okura V. K. (2001). The genome of the natural genetic engineer *Agrobacterium tumefaciens* C58. *Science* 294 2317–2323. 10.1126/science.1066804 11743193

[B54] Yang J. Kloepper J. W. Ryu C. M. (2009). Rhizosphere bacteria help plants tolerate abiotic stress. *Trends Plant Sci.* 14 1–4. 10.1016/j.tplants.2008.10.004 19056309

[B55] Ye W. Shen C. H. Lin Y. Chen P. J. Xu X. Oelmüller R. (2014). Growth promotion-related miRNAs in *Oncidium* orchid roots colonized by the endophytic fungus *Piriformospora indica*. *PLoS ONE* 9:e84920 10.1371/journal.pone.0084920 PMC3883679 24409313

