# Supplementary material for: The Abundance of Endofungal Bacterium Rhizobium radiobacter (syn. Agrobacterium tumefaciens) Increases in Its Fungal Host Piriformospora indica during the Tripartite Sebacinalean Symbiosis with Higher Plants
Source: Front Microbiol. 2017 Apr 13;8:629. doi: 10.3389/fmicb.2017.00629 (PMC5390018; doi:10.3389/fmicb.2017.00629)
Supplement: Supplementary file 1 [file Image_1.PDF]

Supplement Figure 1

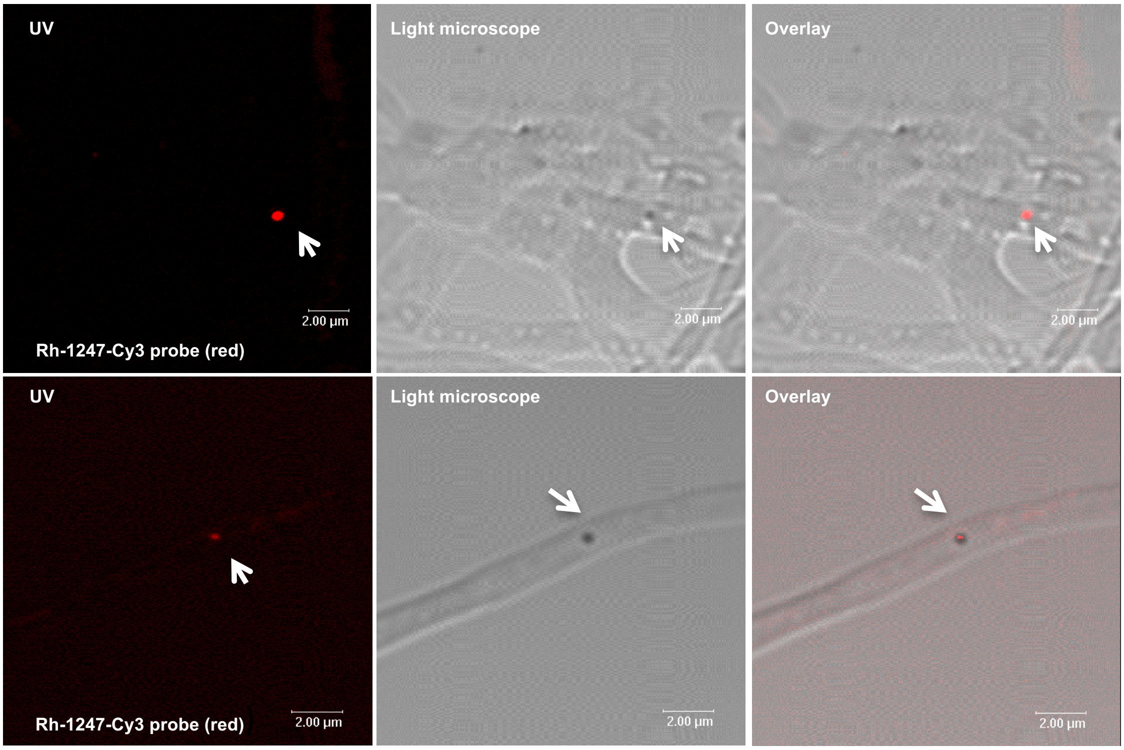

Supplement Figure 1: Detection of endobacteria in *Piriformospora indica* with confocal laser scanning microscopy (CLSM). The fungus was grown in liquid CM medium for three weeks, fixed for FISH, checked with the specific *Rhizobium* probe Rh-1247-Cy3, and analyzed by CLSM. Bacteria (red signal) were detected under UV and light microscope. White arrows point to bacteria.

Supplement Figure 2

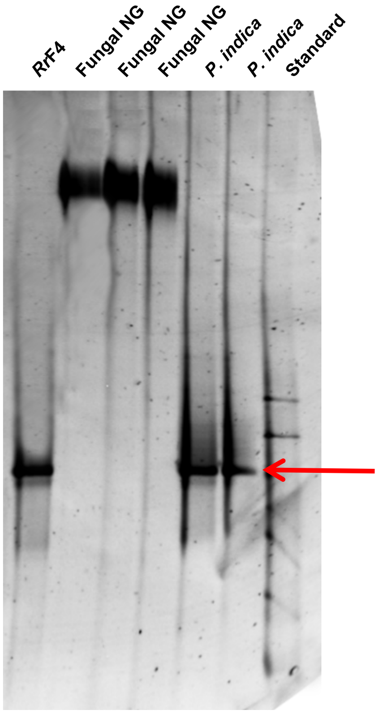

Supplement Figure 2: Detection of endobacteria in *Piriformospora indica* by Denaturing Gradient Gel Electrophoresis (DGGE). A 16S rRNA gene fragment with the same motility in the DGGE gel was amplified from a DNA extract of a pure *Rr*F4 culture and a *P. indica* culture. Fungal NG: Other endophytic fungi.

Supplement Figure 3

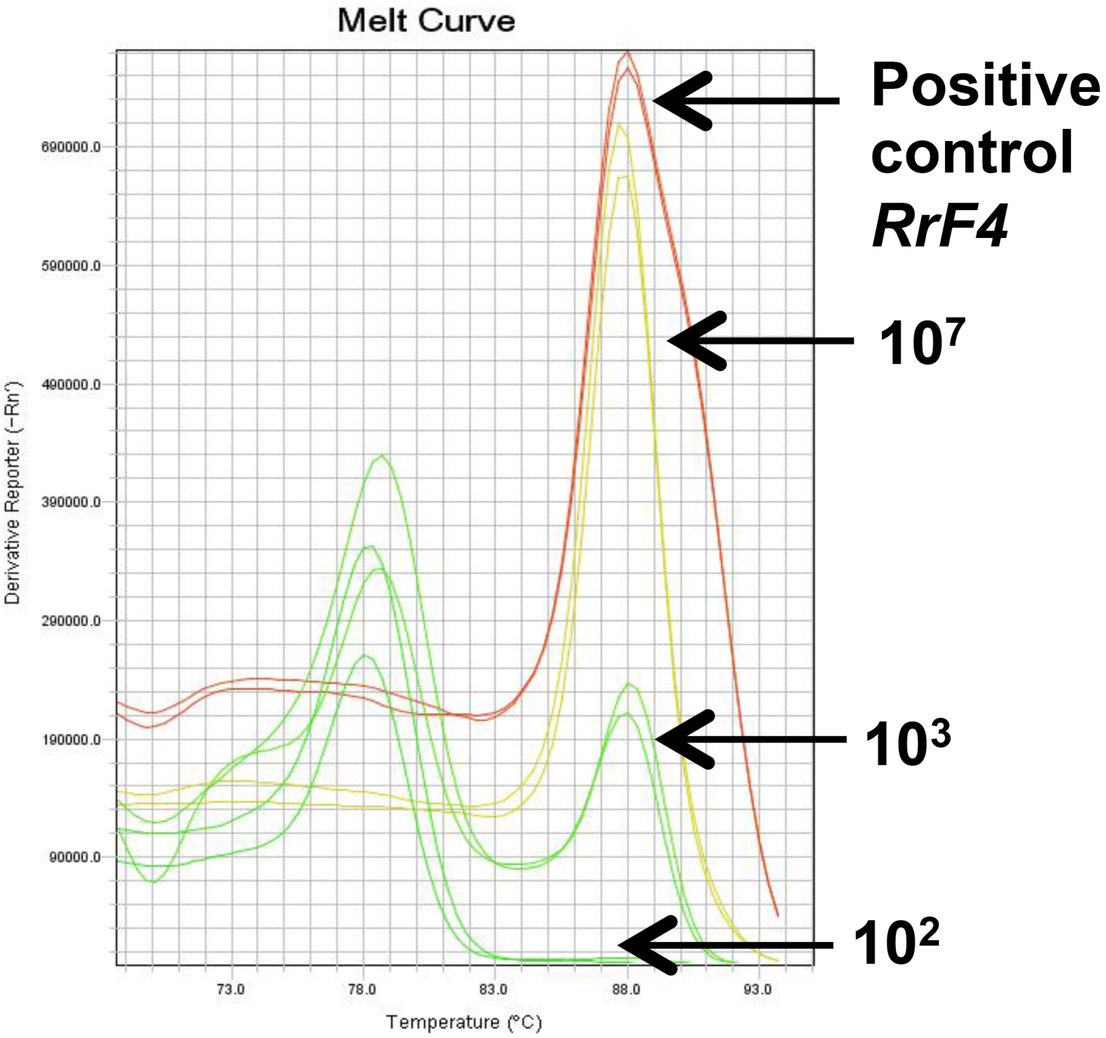

Supplement Figure 3. Melt curve of PCR amplified ITS fragments and primer dimers in qPCR. Three different concentrations of ITS targets (10^7, 10^3 and 10^2 per PCR reaction) and pure *Rr*F4 were used to see the primer efficiency. There was melting peak (T_m) with 10^7 and 10^3 targets at 83ºC as in pure *Rr*F4. There was only a melting peak from primer dimer with 10^2 targets at 78ºC instead of the ITS specific melting peak at 83ºC.
